# P2X7 receptor regulates osteoclast function and bone loss in a mouse model of osteoporosis

**DOI:** 10.1038/s41598-018-21574-9

**Published:** 2018-02-22

**Authors:** Ning Wang, Ankita Agrawal, Niklas Rye Jørgensen, Alison Gartland

**Affiliations:** 1 The Mellanby Centre for Bone Research, Department of Oncology and Metabolism, The University of Sheffield, Sheffield, UK; 2 Department of Clinical Biochemistry, Rigshospitalet, Glostrup, Denmark; 3 OPEN, Odense Patient data Explorative Network, Odense University Hospital/Institute of Clinical Research, University of Southern Denmark, Odense, Denmark

## Abstract

Post-menopausal osteoporosis is a condition that affects millions worldwide and places a huge socio-economic burden on society. Previous research has shown an association of loss of function SNPs in the gene for the purinergic receptor P2X7R with low bone mineral density, increased rates of bone loss and vertebral fractures in post-menopausal women. In this study we use a mouse model of oestrogen deficiency-induced bone loss and the BALB/cJ P2X7R^−/−^ to show that absence of the P2X7R resulted in increased bone loss. Osteoclast precursors were isolated from both BALB/cJ P2X7R^−/−^ and BALB/cJ P2X7R^+/+^ mice and then cultured *in vitro* to form mature resorbing osteoclasts. The BALB/cJ P2X7R^−/−^ derived precursors generated slightly more osteoclasts but with a significant reduction in the amount of resorption per osteoclast. Furthermore, when using modified culture conditions osteoclast activity was additionally increased in the absence of the P2X7R suggest that P2X7R may regulate the lifespan and activity of osteoclasts. Finally using mechanical loading as an anabolic stimulus for bone formation, we demonstrated that the increased oestrogen-deficient bone loss could be rescued, even in the absence of P2X7R. This study paves the way for clinical intervention for women with post-menopausal osteoporosis and P2XR7 loss of function polymorphisms.

## Introduction

Osteoporosis is a disease of bone that affects millions of people worldwide and places a significant burden on the economy. The disease itself has multiple aetiologies, but the largest cause is the loss of oestrogen production in women following the menopause. This is due to oestrogen acting as a regulator of osteoclast activity and life span^1^.

Osteoclasts are the cells responsible for the breakdown or resorption of bone that is required for the normal maintenance and turnover of bone to keep the skeleton healthy^2^. In post-menopausal osteoporosis, osteoclasts are not kept in control by oestrogen and so become overactive which leads to the temporary uncoupling of bone resorption and formation and results in overall net bone loss and disease^3^.

Current treatments for osteoporosis mainly target the osteoclasts in order to prevent this excessive resorption. The main treatments currently are bisphosphonates which work by binding to the bone surface and inhibiting bone resorption by interfering with the osteoclast signalling pathways leading to cell death and thus reduction in resorption and bone loss^4^. One of the problems with bisphosphonate treatment is that whilst they are effective in preventing osteoclastic resorption, they reduce bone formation due to the coupling between resorption and formation. Other options exist for anti-catabolic drugs such as Denosumab, a fully humanised antibody to Receptor activator of nuclear factor-kB ligand (RANKL, a cytokine essential for osteoclast differentiation, activation, and survival)^5^. In contrast, only one anabolic agent is currently available, teriparatide (an analogue of PTH)^6^. However these alternatives also have problems in that they are not efficacious in fracture prevention at numerous sites. Therefore there is a need for alternative pathways in bone resorption to target for the treatment of bone disease.

One such signalling pathway is the purinergic signalling pathway. Purinergic signalling involves the controlled release of extracellular nucleotides from cells and the subsequent binding to cell-surface receptors called purinergic receptors. The receptors are classified into the P2Y G-protein coupled receptors and the P2X ligand gated ion channels^7^. One intriguing P2X receptor is the P2X7 receptor (P2X7R) which, whilst it has similarities to the other P2X receptors, has distinct properties such as the ability to switch from a channel to pore configuration upon higher and longer exposure to its ligands^8^. The P2X7R has been shown to be involved in multiple cellular responses both due to ion channel activation and pore formation. These include release of interleukins and other cellular contents, cell fusion and apoptosis^9^. In the context of bone the P2X7R is known to be expressed by both osteoblasts and osteoclasts and plays a role in modulating the differentiation, function and life span of both cell types^10^. Recent studies from our labs have also demonstrated that loss of function SNPs in the gene for the P2X7R (P2RX7) are associated with low BMD, accelerated bone loss and vertebral fractures in post-menopausal women^11,12^.

In this current study we have used a mouse model of osteoporosis to investigate oestrogen deficient bone loss in the absence of P2X7R and whether a simple anabolic intervention can mitigate this bone loss.

## Results

### Baseline characteristics of female skeletally mature P2X7R^−/−^ mice

In this study we have used BALB/cJ P2X7R^−/−^ as the previously reported P2X7R null mice were generated on strains of mice that have naturally occurring mutations in the P2rx7 that impairs the function of the receptor^13^. BALB/cJ P2X7R^−/−^ mice had significantly lower tibial cortical tissue mineral density (Ct.TMD) (p = 0.0178, Table 1) and significantly higher tibial cortical bone volume (Ct.BV)(p = 0.0418, Table 1) compared to littermate BALB/cJ P2X7R^+/+^ controls. There were no other statistically significant differences in the morphometric indices measured in either the cortical or trabecular compartment (Table 1). Contrary to this, histological analysis of the tibial bone demonstrated significant effects of P2rx7 gene deletion on bone cell numbers and size. In the cortical bone compartment, on the endocortical bone surface the number (N.Oc) and perimeter of osteoclasts (Oc.Pm) was more than threefold higher (p = 0.015 and p = 0.035 respectively, Table 1), whilst there were half the number of osteoblasts (N.Ob) (p = 0.009) per mm of BALB/cJ P2X7R^−/−^ mice compared to littermate BALB/cJ P2X7R^+/+^ controls. Similarly, in the trabecular bone compartment the osteoclast number and perimeter was two-fold higher (p < 0.001 and p = 0.003 respectively, Table 1) on the trabecular surface of BALB/cJ P2X7R^−/−^ mice compared to littermate BALB/cJ P2X7R^+/+^ controls. Whilst there was a trend towards a reduction in the number of osteoblasts on the trabecular surface in the BALB/cJ P2X7R^−/−^ mice, only the reduction in osteoblast perimeter (Ob.Pm) reached statistical significance (p = 0.041, Table 1).

**Table 1:** Quantitative results of the morphometric tibia bone parameters for BALB/cJ P2X7R^−/−^ and littermate BALB/cJ P2X7R^+/+^ at 4 months of age.

	P2X7R^+/+^ N = 9	P2X7R^−/−^ N = 7	Mean difference	p-value
Ct.TMD (g/cm^3^)	1.506 ± 0.01	1.453 ± 0.02	**↓**	**0**.**0178**
Ct.BV (mm^3^)	0.8175 ± 0.014	0.8727 ± 0.036	**↑**	**0**.**0418**^**a**^
Ct.Th (mm)	181.6 ± 1.5	179.4 ± 6.1		0.6932
TMD (g/cm^3^)	1.262 ± 0.008	1.258 ± 0.011		0.7611
BV/TV	8.595 ± 0.427	9.422 ± 0.823		0.3574
Tb.Th (mm)	0.0500 ± 0.0005	0.0512 ± 0.0010		0.2865
Tb.N (1/mm)	1.717 ± 0.0836	1.835 ± 0.1424		0.4660
Tb.Pf (1/mm)	24.36 ± 0.80	24.11 ± 1.50		0.8787
Tb.Sp (mm)	0.3243 ± 0.0131	0.2993 ± 0.0213		0.3114
SMI	2.122 ± 0.047	2.129 ± 0.073		0.9360
Endo. N.Ob/B Pm	29.34 ± 5.7	15.32 ± 3.4	**↓**	**0**.**009**^**b**^
Endo Ob.Pm/B Pm	0.3213 ± 0.041	0.2393 ± 0.056		0.122^**b**^
Endo. N.Oc/B Pm	0.1603 ± 0.1035	0.5692 ± 0.0925	**↑**	**0**.**015**^**b**^
Endo Oc.Pm/B Pm	0.004975 ± 0.0031	0.01904 ± 0.0046	**↑**	**0**.**035**^**b**^
Tb. N.Ob/B Pm	3.0497 ± 0.9986	1.2951 ± 0.5129		0.098^b^
Tb Ob.Pm/B Pm	0.0553 ± 0.01989	0.0153 ± 0.00457	**↓**	**0**.**041**^**b**^
Tb. N.Oc/B Pm	2.9266 ± 1.032	6.6056 ± 0.5384	**↑**	**<0**.**001**^**b**^
Tb Oc.Pm/B Pm	0.0807 ± 0.02881	0.1705 ± 0.01732	**↑**	**0**.**003**^**b**^
N.Ad/Ma.Ar (mm^2^)	12.93 ± 8.816	18.27 ± 5.508		0.505^b^

Values are mean ± SEM.

Ct. TMD: Cortical tissue mineral density, Ct.BV: Cortical bone volume, Ct.Th: Cortical thickness, TMD: Tissue mineral density, BV/TV: Bone volume fraction, Tb.Th: Trabecular thickness, Tb.N: Trabecular number, Tb.Pf: Trabecular pattern factor, Tb. Sp: Trabecular Separation, SMI: Structure Model Index, Endo. N.Ob/B Pm: Osteoblast number per mm endocortical bone surface, Endo Ob.Pm/B Pm: Percentage endocortical surface occupied by osteoblasts, Endo. N.Oc/B Pm: Osteoclast number per mm endocortical bone surface, Endo Oc.Pm/B Pm: Percentage endocortical surface occupied by osteoclasts, Tb. N.Ob/B Pm: Osteoblast number per mm trabecular bone surface, Tb Ob.Pm/B Pm: Percentage trabecular surface occupied by osteoblasts, Tb. N.Oc/B Pm: Osteoclast number per mm trabecular bone surface, Tb Oc.Pm/B Pm: Percentage trabecular surface occupied by osteoclasts, N.Ad/ Ma.Ar (mm^2^): The number of adipocyte per mm^2^ bone marrow area. Data was analysed using either an unpaired t-test or ^a^Mann-Whitney test, or ^b^Univariate Analysis of Variance as appropriate for the experimental design and data set.

### Absence of the P2X7R alters bone loss in a mouse model of osteoporosis

**Figure 1:**
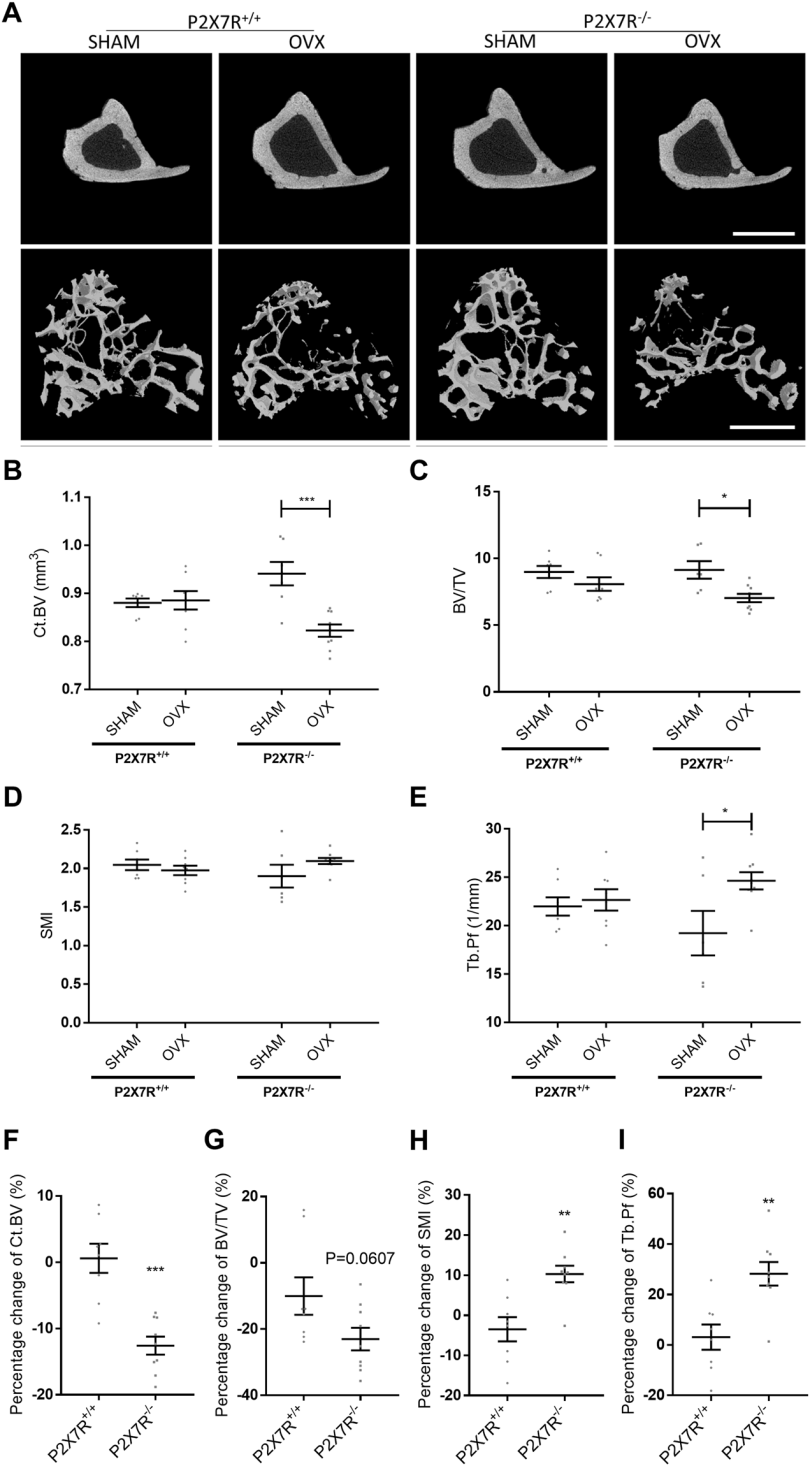
Absence of the P2X7R alters bone loss in a mouse model of osteoporosis. Sixteen week old, virgin, female BALB/cJ P2X7R^+/+^ and BALB/cJ P2X7R^−/−^ mice were bilaterally ovariectomized (OVX) or ovaries were exposed without removal (SHAM). Six weeks after surgery, proximal ends of tibiae were μCT scanned. (**A**) Representative μCT images of the tibial cortical bone is shown in the top panel and 3D models of the trabecular bone, built from a region of 1.0 mm thick trabecular bone 0.2 mm below the growth plate in the tibia, is shown in the bottom panel. Scale bar = 1 mm. The bone structural changes were characterized by measuring parameters including (**B**) cortical bone volume (Ct.BV), (**C**) trabecular bone volume fraction (BV/TV), (**D**) trabecular Structure Model Index (SMI) and (**E**) trabecular pattern factor (Tb.Pf). (**H** to **I**) To further determine the differential respond to OVX between the two strains, the percentage changes in the bone architecture after OVX (% of (1-OVX/mean of SHAM) were also compared. All values are mean ± SEM, n = 6–9 mice per group. *p < 0.05, **p<0.01, ***p<0.001, indicate statistically significant differences, using unpaired parametric t-test or non-parametric Mann-Whitney test as appropriate.

In an *in vivo* model of post-menopausal bone loss, bone architectural changes were examined and characterised using μCT (Fig. 1A). Successful OVX was confirmed by analysis of the uterine weights; both BALB/cJ P2X7R^+/+^ and BALB/cJ P2X7R^−/−^ OVX mice had a significant reduction in the uterine weights compared to their respective SHAM controls (p = 0.0003 and p = 0.0006 respectively, Table 2). BALB/cJ P2X7R^−/−^ mice had a much greater response to ovariectomy surgery (OVX) than BALB/cJ P2X7R^+/+^ mice with increased cortical (Ct.BV) and trabecular (BV/TV) bone loss compared to the SHAM operated mice (Fig. 1B, p = 0.0004 and Fig. 1C, p = 0.0145 respectively; Table 2). Differences in the change to of the Structure Model Index (SMI) and significant changes in the pattern (Tb.Pf) of trabeculae of the OVX BALB/cJ P2X7R^−/−^ mice were also detected compared to SHAM operated mice (Fig. 1D,E respectively, Table 2). To further confirm whether P2X7R^+/+^ and P2X7R^−/−^ mice respond differently to OVX, we have compared the percentage changes in the bone architecture after OVX between the two strains. BALB/cJ P2X7R^−/−^ mice had 13% more bone loss in both the cortical (p = 0.0001, Fig. 1F) and trabecular (p = 0.0607, Fig. 1G) bone compartments, but 13% greater increase in SMI (p = 0.0022, Fig. 1H) and 25% greater increase in Tb.Pf (p = 0.0016, Fig. 1I) after OVX, compared to BALB/cJ P2X7R^+/+^ mice.

**Table 2:** Quantitative results of tibia bone parameters for BALB/cJ P2X7R^−/−^ and littermate BALB/cJ P2X7R^+/+^ following OVX or SHAM surgery.

	P2X7R^+/+^		p-value	P2X7R^−/−^		p-value
	SHAM N = 7	OVX N = 8		SHAM N = 6	OVX N = 9	
Uterine weight (mg)	78.90 ± 9.1	19.30 ± 4.0	**0**.**0003**	77.19 ± 5.9	15.44 ± 2.2	**0**.**0006**
Ct.TMD (g/cm^3^)	1.51 ± 0.01	1.48 ± 0.01	0.0432	1.47 ± 0.01	1.50 ± 0.01	0.2350
Ct.BV (mm^3^)	0.88 ± 0.01	0.89 ± 0.02	0.6126^a^	0.94 ± 0.02	0.82 ± 0.01	**0**.**0004**
TMD (g/cm^3^)	1.23 ± 0.01	1.25 ± 0.01	0.3189	1.24 ± 0.01	1.26 ± 0.01	0.2269
BV/TV	8.98 ± 0.45	8.08 ± 0.51	0.2113	9.13 ± 0.66	7.03 ± 0.31	**0**.**0145**
Tb.Th (mm)	0.052 ± 0.001	0.049 ± 0.001	0.1206^a^	0.053 ± 0.002	0.049 ± 0.001	0.0574
Tb.N (1/mm)	1.72 ± 0.09	1.65 ± 0.12	0.6292	1.73 ± 0.12	1.43 ± 0.06	**0**.**0268**
Tb.Pf (1/mm)	21.98 ± 0.95	22.66 ± 1.09	0.6533	19.22 ± 2.30	24.64 ± 0.89	**0**.**0257**
Tb.Sp (mm)	0.32 ± 0.01	0.34 ± 0.02	0.2407	0.33 ± 0.01	0.37 ± 0.02	0.1169
SMI	2.047 ± 0.068	1.976 ± 0.062	0.4514	1.901 ± 0.147	2.097 ± 0.039	0.2550

Values are mean ± SEM.

Ct.TMD: Cortical tissue mineral density, Ct.BV: Cortical bone volume. TMD: Tissue mineral density, BV/TV: Bone volume fraction, Tb.Th: Trabecular thickness, Tb.N: Trabecular number, Tb.Pf: Trabecular pattern factor, Tb.Sp: Trabecular Separation, SMI: Structure Model Index, Data was analysed using either an unpaired t-test or ^a^Mann-Whitney test as appropriate for the data sets.

### Absence of the P2X7R renders osteoclast precursor cells sensitive to exogenous stimuli

**Figure 2:**
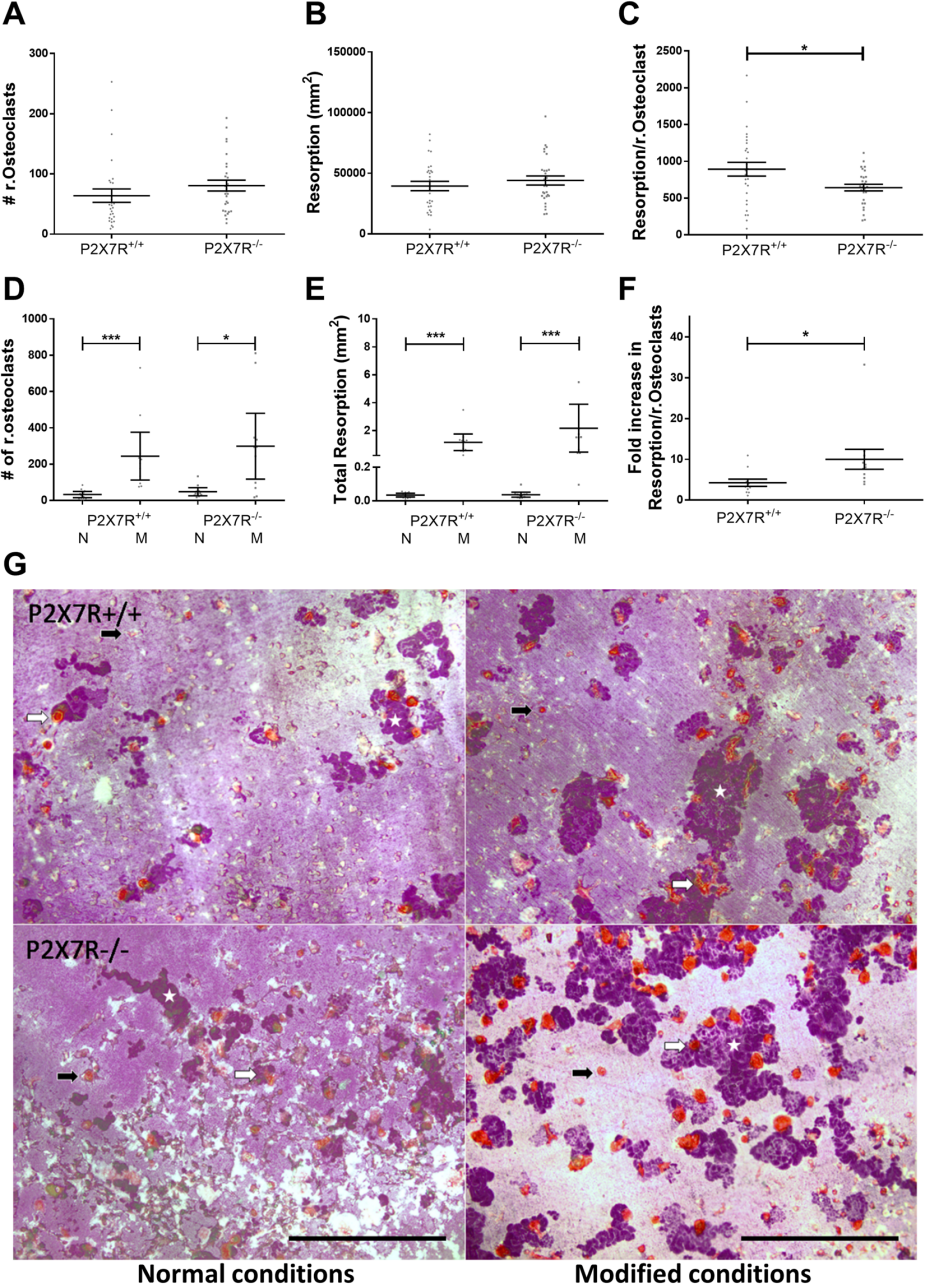
Absence of the P2X7R renders osteoclast precursor cells sensitive to exogenous stimuli. Precursor cells from bone marrow aspirates of BALB/cJ P2X7R^+/+^ and BALB/cJ P2X7R^−/−^ were differentiated on dentine and TRAP stained. In normal (N) conditions the (**A**) total number of resorbing osteoclasts, (**B**) total resorption and (**C**) the resorptive ability (resorption/resorbing osteoclast) was determined. In modified (M) conditions the (**D**) total number of resorbing osteoclasts, (**E**) total resorption and (**F**) response to modified conditions on the resorptive ability of osteoclasts (expressed as a fold change of the mean resorption/resorbing osteoclast of the normal conditions (N) in either genotype) was determined. Values are mean ± SEM, n = 3 repeat cultures containing a total of 11 dentine discs. *p<0.05 **p<0.01, ***p<0.001, unpaired parametric t-test or non-parametric Mann-Whitney test as appropriate. (**G**) Representative images show non-resorbing (black arrows) and resorbing (white arrows) osteoclasts and resorption trails (white stars) excavated by the cells on dentine in both normal and modified conditions. Scale bar = 500 µm.

The ability of bone marrow precursor cells to form osteoclasts from BALB/cJ P2X7R^−/−^ mice was compared to BALB/cJ P2X7R^+/+^ mice. Cultures of cells isolated from BALB/cJ P2X7R^−/−^ mice had slightly more osteoclasts but a significantly reduced amount of resorption per osteoclast (p = 0.016) (Fig. 2A+ C). This data is consistent with the *in vivo* data above – increased numbers of osteoclast on the trabecular bone surface without any changes in trabecular bone volume as detected by μCT. We next modified the culture conditions to determine whether the P2X7R^−/−^ osteoclast precursors would be sensitive to changes in exogenous stimuli. We used phenol red free medium (as phenol red acts as a weak oestrogen^14^) and charcoal stripped serum to remove lipophilic, biologically active compounds from the serum. Under these conditions, cells from both BALB/cJ P2X7R^+/+^ and BALB/cJ P2X7R^−/−^ mice have significantly and substantially increased numbers of osteoclasts and resorption (Fig. 2D,E,G). However, the fold increase in the resorptive capacity of osteoclasts in these conditions was significantly higher in cells from the BALB/cJ P2X7R^−/−^ mice compared to the cells from the BALB/cJ P2X7R^+/+^ mice (10 fold increase *cf* 4 fold increase respectively, p = 0.018, Fig. 2F). This data further supports the *in vivo* data above that osteoclast activity is additionally increased in the absence of any osteoclastogenic exogenous stimuli and the P2X7R, suggesting that P2X7R may regulate the lifespan and activity of osteoclasts.

### Mechanical loading rescues bone loss in a mouse model of osteoporosis even in the absence of the P2X7R

**Figure 3:**
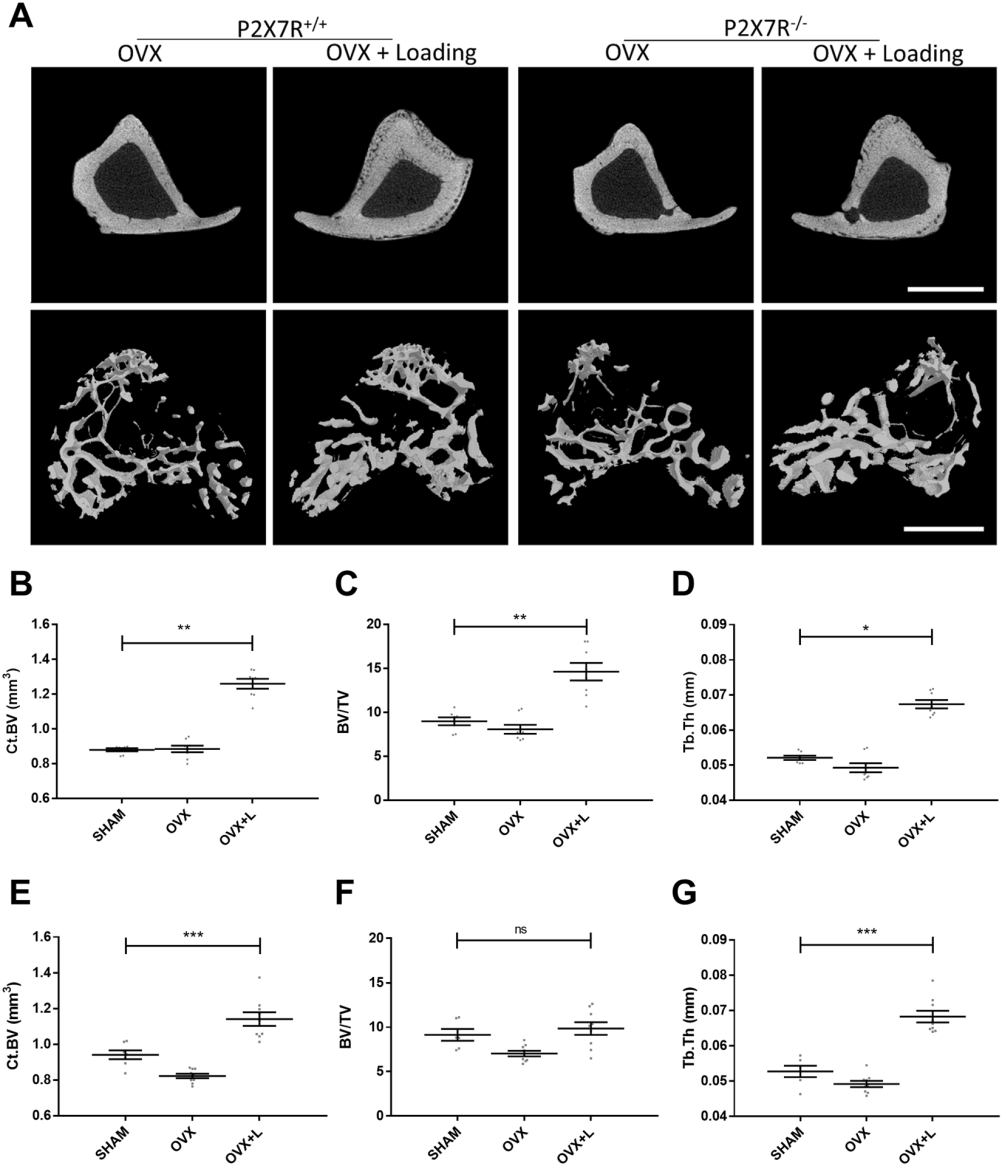
Mechanical loading rescues bone loss in a mouse model of osteoporosis even in the absence of the P2X7R. BALB/cJ P2X7R^+/+^ and BALB/cJ P2X7R^−/−^ OVX and SHAM mice underwent mechanical loading 4 weeks after surgery. A 13.5 N dynamic load was superimposed onto a 0.5 N pre-load at rate of 160,000 N/s. Forty trapezoidal-waveform load cycles (0.2 s hold at 14 N) with 10 s interval between each cycle were applied to mice tibiae, three times a week for 2 weeks. The proximal ends of tibiae were μCT scanned and representative μCT images of the tibial cortical bone are shown in the top panel in **A**, and three-dimension models of the trabecular bone, built from a region of 1.0 mm thick trabecular bone 0.2 mm below the growth plate of tibiae, are shown in the bottom panel in **A**. Scale bar = 1 mm. Bone architectural changes were characterized by measuring structural parameters including cortical bone volume (Ct.BV), trabecular bone volume/tissue volume (BV/TV), and trabecular thickness (Tb.Th) in both (**B**,**C**,**D**) BALB/cJ P2X7R^+/+^ and (**E**,**F**,**G**) BALB/cJ P2X7R^−/−^ mice. All values are mean ± SEM, n = 6–9 mice per group. *p < 0.05, **p < 0.01, **p < 0.001, 1-way ANOVA with multiple comparison post-test appropriate to the data set.

Mechanical loading of bone is a potent anabolic stimulus for new bone formation^15^ that is increasingly becoming an attractive intervention, both as a preventative measure and as a treatment option for osteoporosis^16,17^, either alone or in combination with drugs that target the bone remodelling pathway^18,19^. Therefore, we next tested whether mechanical loading would rescue the bone loss in our mouse models of osteoporosis. Loading of the tibia significantly induced new bone formation in OVX-BALB/cJ P2X7R^+/+^ mice (Fig. 3A), with significant increases in cortical bone volume (Fig. 3B, p < 0.001), trabecular bone volume (Fig. 3C, p < 0.01) and trabecular thickness (Fig. 3D, p < 0.05) to values above that of SHAM-BALB/cJ P2X7R^+/+^ mice. One of the key early events in the response of cells, including those of bone, to mechanical loading is the release of ATP and activation of purinergic receptors. Indeed, a previous study demonstrated that P2X7R null mice had up to 73% reduced sensitivity to mechanical loading, suggesting a key role in mechanosensitivity^20^. Surprisingly, loading of OVX-BALB/cJ P2X7R^−/−^ mice still rescued the increased bone loss seen after OVX surgery (Fig. 3A), with increases in cortical bone volume (Fig. 3E, p < 0.001), trabecular bone volume (Fig. 3F,) and trabecular thickness (Fig. 3G, p < 0.001) all to at least the level of SHAM-BALB/cJ P2X7R^−/−^ mice.

## Discussion

In this study we demonstrate that the P2X7R protects against bone loss in a mouse model of post-menopausal osteoporosis. At sixteen weeks of age we found that the BALB/cJ P2X7R^−/−^ mouse had reduced cortical BMD, increased cortical volume but similar trabecular architecture and values as compared to BALB/cJ P2X7R^+/+^ mice using μCT analysis. This baseline phenotype is consistent with the previously reported bone phenotype of the P2X7R null mice^21,22^ suggesting that deletion of the P2X7R only has a mild effect on bone under physiological conditions. Structural changes in bone are the results of altered cell numbers and activity in one or more of the cell types. Upon histological examination of the mice, we found that the BALB/cJ P2X7R^−/−^ mice had significantly fewer osteoblasts and significantly more osteoclasts covering the cortical bone surface compared to BALB/cJ P2X7R^+/+^ mice. This imbalance in bone cell number in favour of osteoclasts would lead to more bone resorption and could possibly account for the reduced cortical BMD. We also found that the number of osteoclasts in the trabecular bone were increased and was accompanied by a reduction in osteoblast coverage. However, these differences did not lead to changes in trabecular architectural indices as measured by μCT. This could be due to the higher turnover rate that is observed in this bone compartment masking any subtle effects of P2X7R deletion. Alternatively, the effect of P2X7R signalling may be site specific; this is not an uncommon phenomenon and parallels the effects of PTH on bone^23^. A potential criticism of our mouse model is that a splice variant of P2X7R, P2X7R(k), has previously been found in the founder Glaxo P2X7R^−/−^ mice in various tissues^24^ and in osteoclasts cultures at the mRNA level but not at the level of the protein^25^. However, although we did find expression of the P2X7R(k) variant in the highly heterogeneous bone marrow populations, we did not find any expression of the P2X7R(k) variant in mature resorbing osteoclasts or primary osteoblasts (see Supplementary Data Fig. 1), confirming that this model is indeed a knock out in these cells and ruling out any contribution of a functional P2X7(k) variant in bone cells to the results obtained with the BALB/cJ P2X7R^−/−^ mice. These findings suggest that deletion of the P2X7R results in an osteopenic-like bone phenotype under normal physiological conditions and is consistent with the association of low BMD and fracture risk in subjects with loss of function polymorphisms in P2RX7^11,26^.

We have also previously reported that loss of function P2RX7 polymorphisms are also associated with increased rates of bone loss, vertebral fractures and effect of oestrogen treatment in post-menopausal women^11,12,27^, so we wanted to determine whether P2X7R deletion would lead to increased bone loss following oestrogen loss in our mouse model. We confirmed successful OVX surgery by the lack of any ovaries post-mortem in both strains of mice. Six weeks post-OVX the BALB/cJ P2X7R^−/−^ mice had significant bone loss, particularly with a greater loss of cortical bone, than in the BALB/cJ P2X7R^+/+^ mice. At this time point the BALB/cJ P2X7R^+/+^ mice had already started to restore the bone loss by compensatory mechanisms, as has been suggested in previous studies using rodent models^28–30^. When the individual data for the BALB/cJ P2X7R^+/+^ mice is taken into account, two mice in the OVX group have recovered their trabecular bone loss (Fig. 1C). These mice can be identified as statistically significant outliers using the ROUT (Q = 2%) analysis and should these two outliers be removed from the data set, then the OVX group shows a statistically significant 18.4% reduction in BV/TV compared to SHAM control (p = 0.0082).

In addition to changes in bone loss post-OVX, the BALB/cJ P2X7R^−/−^ mice also have significantly altered bone microarchitecture indices compared to BALB/cJ P2X7R^+/+^ mice. The most intriguing being the significantly increased trabecular pattern factor (Tb.Pf) and Structure Model Index (SMI), which indicate less well-connected and more rod-like structured trabecular bone in P2X7R^−/−^ mice, in contrast to the well-connected and flat structure observed in normal healthy animals. This type of bone microarchitecture change has been suggested to be associated with abnormally enhanced activities of osteoclasts which controls the trabecular connection and the conversion of trabecular from plate elements to rod elements^31,32^. OVX removes oestrogen which normally controls osteoclast life span and apoptosis. The P2X7R has been linked to cell death and apoptosis in a number of cell lines including osteoclasts^27,33,34^, and in an intact mouse could provide an alternative level of control of osteoclast numbers in the absence of oestrogen. However, in the BALB/cJ P2X7R^−/−^ mouse post-OVX, both oestrogen- and P2X7R-induced osteoclast apoptosis are missing potentially leading to increased longevity of these cells, sustained bone resorption and thus net bone loss. This is consistent with the previously reported observation of reduced apoptosis following ATP stimulation of osteoclasts generated from women with loss-of function P2RX7 SNPs^27^.

Additional evidence for the role of the P2X7R in osteoclast survival and function is provided by the *in vitro* studies. In keeping with previously reported literature^21,35^, monocytes from the BALB/cJ P2X7R^−/−^ mice were able to fuse and form multinucleated osteoclasts. This rules out a major critical role for the P2X7R in osteoclast cell fusion, as had previously been speculated. Furthermore, in the cultures from the BALB/cJ P2X7R^−/−^ mice, the number of osteoclasts formed were not significantly different from BALB/cJ P2X7R^+/+^ cultures, again consistent with previous studies. We did note however, that whilst the overall resorption was also unaffected, the amount of resorption per osteoclast was significantly reduced. This suggests that whilst the lack of P2X7R does not affect formation and fusion of osteoclasts, it does have an effect on their functional activity. This would suggest that the increased numbers of osteoclasts observed *in vivo* may well be a compensatory mechanism for their reduced activity.

As it is difficult to determine the activity of osteoclasts *in vivo* via histology, we investigated this *in vitro* by culturing monocytes in altered culture medium in an attempt to mimic the altered paracrine conditions in the OVX mouse. We used phenol red free media and heat inactivated charcoal stripped FBS as this has previously been used as a model of oestrogen-depletion *in vitro*^36^. When the cells were cultured this way, BALB/cJ P2X7R^−/−^ cultures had a greater increase in the number of resorbing osteoclasts, total resorption and the amount of resorption per osteoclast than BALB/cJ P2X7R^+/+^ cultures. Whilst these altered cell culture conditions, especially the charcoal stripped FBS, will also remove biologically active lipophilic molecules which may also affect osteoclast formation and function, this data suggests that absence of P2X7R renders the cells more sensitive to changes in exogenous stimuli, including oestrogen, that increases the survival and thus overall resorption of osteoclasts.

A potential anabolic treatment for bone loss is exercise due to increased mechanical loading. It has previously been suggested that the P2X7R plays an important role in the transduction of mechanical load^20^. Therefore we wanted to determine what would be the effect of mechanical loading on our post-menopausal model. In both the BALB/cJ P2X7R^−/−^ and BALB/cJ P2X7R^+/+^ mice, 2 weeks of mechanical loading significantly increased the cortical and trabecular bone volume to the equivalent or greater value than the SHAM operated controls. This data confirms that the BALB/cJ P2X7R^−/−^ mice are still capable of responding to mechanical loading. Trabecular thickness was also increased in both strains, suggesting increased osteoblast activity. The discrepancy with the previous literature that demonstrated a reduced response to mechanical load in another P2X7R^−/−^ mouse model could be due to the different types of mechanical loading used. Li *et al*. used lower forces and only 3 consecutive days of mechanical loading^20^ whilst we loaded every other day for 2 weeks. In addition, in our current study the BALB/cJ P2X7R^−/−^ mice also have increased osteoclast activity and active resorption due to the response to OVX - coupled with an activated osteogenic response from loading resulted in a net effect of a greater level of bone formation.

In summary, we have confirmed that loss of the P2X7R alters oestrogen-deficient bone loss, as seen in post-menopausal women with loss of function polymorphism in the P2X7R. This effect is likely to be due to the increased activity and life span of osteoclasts in the absence of both oestrogen and P2X7R. The observation that the anabolic stimulus of mechanical loading rescued the increased bone loss in the absence of P2X7R suggests a potential clinical intervention to help treat post-menopausal osteoporosis in women with loss of function P2RX7 polymorphisms.

## Methods

### Mice

P2X7R^−/−^ mice^37^ were backcrossed onto the BALB/cJ background as previously described^22^. Sixteen week old BALB/cJ P2X7R^−/−^ and BALB/cJ P2X7R^+/+^ littermate control mice were housed in the same environmentally controlled conditions with a 12hr light/dark cycle at 22 °C and free to access *2018 Teklad* Global 18% Protein Rodent Diet containing 1.01% Calcium (Harlan Laboratories, UK) and water *ad libitum* in RB-3 cages. All procedures complied with the UK Animals (Scientific Procedures) Act 1986 and were reviewed and approved by the local Research Ethics Committee of The University of Sheffield (Sheffield, UK).

### OVX surgery

Sixteen week old, virgin, female BALB/cJ P2X7R^+/+^ and BALB/cJ P2X7R^−/−^ mice (n = 6–9/group) were bilaterally ovariectomized (OVX) or ovaries were exposed without removal (SHAM). OVX and SHAM surgery were performed as previously described^38^. Briefly, mice were anesthetized with 1.5–4% isoflurane in oxygen for surgery. The back of each mouse was shaved and surrounding area was cleaned with 70% ethanol. A dorsal incision was made through the skin in the region between the dorsal hump and the base of the tail. The ovaries, surrounding ovarian fat pad, and part of the uterine horns under the abdominal wall were removed. For SHAM surgery, the ovaries and proximal parts of the uterine horns were exteriorised briefly then returned to the abdominal cavity before wound closure.

### Mechanical loading *in vivo*

BALB/cJ P2X7R^+/+^ and BALB/cJ P2X7R^−/−^ OVX and SHAM mice underwent mechanical loading 4 weeks after surgery. A 13.5 N dynamic load was superimposed onto a 0.5 N pre-load at the rate of 160,000 N/s. Forty trapezoidal-waveform load cycles (0.2 s hold at 14 N) with 10 s interval between each cycle were applied to mice tibiae, three times a week for 2 weeks, mice were then euthanized on day 14^39^. Both tibiae were dissected and fixed in 70% ethanol for μCT. The contra-lateral non-loaded limb (right tibia) was treated as internal control for loading (the functional adaption in both cortical and trabecular bone being controlled locally and confined to the loaded bones^40,41^).

### μCT

Fixed tibiae were scanned using a SkyScan 1172 desktop μCT machine at a resolution of 4.3μm with the X-ray source operating at 50 kV, 200μA and using a 0.5 mm aluminium filter. Two-dimensional μCT images were captured and reconstructed by Skyscan NRecon software at threshold of 0.0–0.16 for tibia proximal end. For the tibia proximal end scan, trabecular morphometry was characterized by measuring structural parameters in a 1.0 mm thick trabecular region which is 0.2 mm below the growth plate. Cortical morphometry was quantified from the cortical regions located in the proximal 20% (1.0 mm thick, 1.0 mm below the growth plate). Bone tissue mineral densities (TMD) equal to grams of hydroxylapatite per cube centimetre were calculated based on image greyscale with the following equation: TMD = (0.012 × greyscale value) − 0.296^42^. Nomenclature and symbols were used to describe the μCT derived bone morphometries according to the recommendations from Bouxsein *et al*., 2010^43^.

### Bone marrow osteoclast cultures

Osteoclasts were derived from the mononuclear hematopoietic cell population from the long bone marrow of 12 week old female mice. The bone marrow of limbs was flushed out by PBS using a syringe with 25-gauge needle. Cells were harvested by centrifugation and resuspended in the selection medium (α MEM + GLUTAMAX™ (Gibco), 100 Units/mL Penicillin and 100 μg/mL Streptomycin, 10% FBS, and 30ng/ml M-CSF. Cells were then transferred into a T75 flask and incubated for 24 hours at 37°C, 5% CO_2_ to allow the attachment of stromal cells. Non-adherent cells were collected by centrifugation and resuspended in growth medium (α MEM + GLUTAMAX™ containing 100 Units/mL Penicillin and 100 μg/mL Streptomycin, 10% FBS, 150ng/ml M-CSF, and 30ng/ml murine RANKL (R&D System)). Cells were then seeded onto dentine disks (generated in-house) in 96-well plates at density of 0.5 × 10^6^ cells per well and incubated overnight at 37 °C, 7% CO_2_ to allow the attachment of osteoclast precursor cells. The wells were washed once and cells were cultured at 37 °C, 7% CO_2_ with the medium being replaced every 2–3 days. Cells on dentine disks were cultured for 17 days to allow time for resorption, fixed in ice cold 10% buffered formalin, TRAP stained and counterstained by Gill’s haematoxylin^44^. The number of resorbing osteoclasts (defined as a TRAP positive cell in or in close proximity to resorption pits) and the amount of resorption (area of excavated surface) per dentine disk were quantified.

### Modified bone marrow osteoclast cultures

Mononuclear hematopoietic cell population from the long bone marrow of 12 week old female mice were harvested and cultured as described above at 37 °C, 7% CO_2_ with the medium being replaced every 2–3 days. For modified conditions, the growth medium was phenol red free α MEM containing 100 Units/mL Penicillin and 100 μg/mL Streptomycin, 10% Charcoal stripped FBS, 150ng/ml M-CSF, and 30ng/ml murine RANKL (R&D System). Dentine disks were cultured for 17 days, TRAP stained^44^ and the number of resorbing osteoclasts and the amount of resorption per dentine disk were quantified as above.

### Statistics

All data are expressed as mean ± SEM. Data was tested for normality using the Kolmogorov-Smirnov test and then statistical significance was tested using either an unpaired parametric t-test, non-parametric Mann-Whitney test, ANOVA or Univariate Analysis of Variance as appropriate for the experimental design using Prism 7 software (GraphPad) and SPSS Statistics 24.

## Electronic supplementary material

Supplementary Information

## References

[1] Kameda T (1997). Estrogen inhibits bone resorption by directly inducing apoptosis of the bone-resorbing osteoclasts. J Exp Med.

[2] Boyle WJ, Simonet WS, Lacey DL (2003). Osteoclast differentiation and activation. Nature.

[3] Vaananen HK, Harkonen PL (1996). Estrogen and bone metabolism. Maturitas.

[4] Russell RG, Watts NB, Ebetino FH, Rogers MJ (2008). Mechanisms of action of bisphosphonates: similarities and differences and their potential influence on clinical efficacy. Osteoporos Int.

[5] McClung MR (2006). Inhibition of RANKL as a treatment for osteoporosis: preclinical and early clinical studies. Curr Osteoporos Rep.

[6] Metcalf LM, Aspray TJ, McCloskey EV (2017). The effects of parathyroid hormone peptides on the peripheral skeleton of postmenopausal women. A systematic review. Bone.

[7] Zimmermann H (2016). Extracellular ATP and other nucleotides-ubiquitous triggers of intercellular messenger release. Purinergic Signal.

[8] Rassendren F (1997). The permeabilizing ATP receptor, P2X7. Cloning and expression of a human cDNA. J Biol Chem.

[9] Bartlett R, Stokes L, Sluyter R (2014). The P2X7 receptor channel: recent developments and the use of P2X7 antagonists in models of disease. Pharmacol Rev.

[10] Agrawal A, Gartland A (2015). P2X7 receptors: role in bone cell formation and function. J Mol Endocrinol.

[11] Gartland A (2012). Polymorphisms in the P2X7 receptor gene are associated with low lumbar spine bone mineral density and accelerated bone loss in post-menopausal women. Eur J Hum Genet.

[12] Jorgensen NR (2012). Single-nucleotide polymorphisms in the P2X7 receptor gene are associated with post-menopausal bone loss and vertebral fractures. Eur J Hum Genet.

[13] Adriouch S (2002). Cutting edge: a natural P451L mutation in the cytoplasmic domain impairs the function of the mouse P2X7 receptor. J Immunol.

[14] Berthois Y, Katzenellenbogen JA, Katzenellenbogen BS (1986). Phenol red in tissue culture media is a weak estrogen: implications concerning the study of estrogen-responsive cells in culture. Proc Natl Acad Sci USA.

[15] Turner CH, Owan I, Alvey T, Hulman J, Hock JM (1998). Recruitment and proliferative responses of osteoblasts after mechanical loading *in vivo* determined using sustained-release bromodeoxyuridine. Bone.

[16] Galloway MT, Jokl P (2000). Aging successfully: the importance of physical activity in maintaining health and function. J Am Acad Orthop Surg.

[17] Rubin C, Turner AS, Bain S, Mallinckrodt C, McLeod K (2001). Anabolism. Low mechanical signals strengthen long bones. Nature.

[18] Braith RW (2007). Comparison of alendronate vs alendronate plus mechanical loading as prophylaxis for osteoporosis in lung transplant recipients: a pilot study. J Heart Lung Transplant.

[19] Braith RW (2003). Resistance exercise training and alendronate reverse glucocorticoid-induced osteoporosis in heart transplant recipients. J Heart Lung Transplant.

[20] Li J, Liu D, Ke HZ, Duncan RL, Turner CH (2005). The P2X7 nucleotide receptor mediates skeletal mechanotransduction. J Biol Chem.

[21] Gartland A (2003). Multinucleated osteoclast formation *in vivo* and *in vitro* by P2X7 receptor-deficient mice. Crit Rev Eukaryot Gene Expr.

[22] Syberg S (2012). Genetic Background Strongly Influences the Bone Phenotype of P2X7 Receptor Knockout Mice. J Osteoporos.

[23] Iida-Klein A (2002). Anabolic action of parathyroid hormone is skeletal site specific at the tissue and cellular levels in mice. J Bone Miner Res.

[24] Nicke A (2009). A functional P2X7 splice variant with an alternative transmembrane domain 1 escapes gene inactivation in P2X7 knock-out mice. J Biol Chem.

[25] Hansen RR (2011). P2X7 receptor-deficient mice are susceptible to bone cancer pain. Pain.

[26] Varley I (2016). Functional polymorphisms in the P2X7 receptor gene are associated with stress fracture injury. Purinergic Signal.

[27] Ohlendorff SD (2007). Single nucleotide polymorphisms in the P2X7 gene are associated to fracture risk and to effect of estrogen treatment. Pharmacogenet Genomics.

[28] Sheng ZF (2007). Regionally specific compensation for bone loss in the tibial trabeculae of estrogen-deficient rats. Acta Radiol.

[29] Laib A, Kumer JL, Majumdar S, Lane NE (2001). The temporal changes of trabecular architecture in ovariectomized rats assessed by MicroCT. Osteoporos Int.

[30] Ottewell PD (2014). Zoledronic acid has differential antitumor activity in the pre- and postmenopausal bone microenvironment *in vivo*. Clin Cancer Res.

[31] Hahn M, Vogel M, Pompesius-Kempa M, Delling G (1992). Trabecular bone pattern factor–a new parameter for simple quantification of bone microarchitecture. Bone.

[32] Hildebrand T, Ruegsegger P (1997). Quantification of Bone Microarchitecture with the Structure Model Index. Comput Methods Biomech Biomed Engin.

[33] Modderman WE, Weidema AF, Vrijheid-Lammers T, Wassenaar AM, Nijweide PJ (1994). Permeabilization of cells of hemopoietic origin by extracellular ATP4-: elimination of osteoclasts, macrophages, and their precursors from isolated bone cell populations and fetal bone rudiments. Calcif Tissue Int.

[34] De Marchi E, Orioli E, Dal Ben D, Adinolfi E (2016). P2X7 Receptor as a Therapeutic Target. Adv Protein Chem Struct Biol.

[35] Ke HZ (2003). Deletion of the P2X7 nucleotide receptor reveals its regulatory roles in bone formation and resorption. Mol Endocrinol.

[36] Morita M (2016). Selective Estrogen Receptor Modulators Suppress Hif1alpha Protein Accumulation in Mouse Osteoclasts. PLoS One.

[37] Chessell IP (2005). Disruption of the P2X7 purinoceptor gene abolishes chronic inflammatory and neuropathic pain. Pain.

[38] Govoni KE, Wergedal JE, Chadwick RB, Srivastava AK, Mohan S (2008). Prepubertal OVX increases IGF-I expression and bone accretion in C57BL/6J mice. Am J Physiol Endocrinol Metab.

[39] De Souza RL (2005). Non-invasive axial loading of mouse tibiae increases cortical bone formation and modifies trabecular organization: a new model to study cortical and cancellous compartments in a single loaded element. Bone.

[40] Sugiyama T, Price JS, Lanyon LE (2010). Functional adaptation to mechanical loading in both cortical and cancellous bone is controlled locally and is confined to the loaded bones. Bone.

[41] de Souza RL, Pitsillides AA, Lanyon LE, Skerry TM, Chenu C (2005). Sympathetic nervous system does not mediate the load-induced cortical new bone formation. J Bone Miner Res.

[42] Wang N (2012). Reduced bone turnover in mice lacking the P2Y13 receptor of ADP. Mol Endocrinol.

[43] Bouxsein ML (2010). Guidelines for assessment of bone microstructure in rodents using micro-computed tomography. J Bone Miner Res.

[44] Agrawal A, Gallagher JA, Gartland A (2012). Human osteoclast culture and phenotypic characterization. Methods Mol Biol.

