# Supplementary material for: P2X7 receptor regulates osteoclast function and bone loss in a mouse model of osteoporosis
Source: Sci Rep. 2018 Feb 22;8:3507. doi: 10.1038/s41598-018-21574-9 (PMC5823935; doi:10.1038/s41598-018-21574-9)
Supplement: Supplementary file 1 — Supplementary Information [file 41598_2018_21574_MOESM1_ESM.docx]

**P2X7 receptor regulates osteoclast function and bone loss in a mouse model of osteoporosis.**

Ning Wang, Ankita Agrawal, Niklas Rye Jørgensen and Alison Gartland.

**
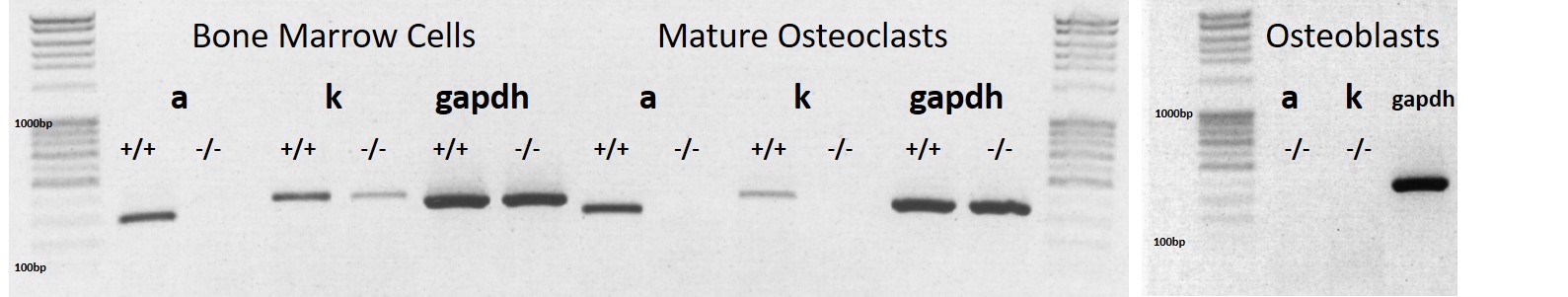
**

**Supplementary Data Figure 1: *P2X7R splice variants are not expressed by bone cells in BALB/cJ P2X7R^-/-^ mice.***

As all the experiments in this current study were performed on BALB/cJ P2X7R KO mice generated from backcrossing the original C57BL/6J P2X7R KO mouse from GlaxoSmithKline we wanted to rule out any contribution of a functional P2X7(k) variant to the results obtained with the BALB/cJ P2X7R^-/-^ mice. Using primers specific for the P2X7R(a) or P2X7R(k) we detected mRNA transcripts for both splice variants in heterogeneous populations of bone marrow cells isolated from the limbs of BALB/cJ P2X7R^+/+^ mice. We could not detect the P2X7R(a) in bone marrow cells isolated from BALB/cJ P2X7R^-/-^ mice, but did detect transcripts for the P2X7R(k). We also detected transcripts for the P2X7R(a) and P2X7R(k) in mRNA from mature osteoclasts grown on dentine disks isolated from BALB/cJ P2X7R^+/+^ mice. In agreement with previous reports we could not detect any transcripts for either P2X7R(a) or (k) splice variant in mRNA from mature osteoclasts grown on dentine disks isolated from BALB/cJ P2X7R^-/-^ mice. In addition, neither P2X7R(a) or (k) splice variant was detected in primary osteoblasts isolated from P2X7R^-/-^ mouse calvarias. Therefore, these results confirm deletion of this receptor in bone cells and would not compromise the bone phenotype related investigations.

*Supplementary Methods: RT-PCR expression of P2X7(a) and P2X7(k) receptor splice variants.*

Bone marrow from long bone and mature osteoclasts cultured on dentine discs (isolated and cultured as described in the main text) were used for RNA extraction. Discs were washed briefly in PBS at day 17 and cells were lysed in TRI Reagent®. For primary osteoblast culture, primary osteoblasts were isolated from 2-day old P2X7R^-/-^ mice calvaria and cultured in DMEM© GLUTAMAX medium with sodium pyruvate, 100 Units/mL Penicillin and 100 μg/mL Streptomycin and 10% foetal calf serum (FCS). The cells were incubated in 37°C incubator with 5% CO2 until confluent. The confluent cells were cultured in medium with differentiation factors (10nM Dexamethasone, 50μg/mL L-Ascorbate Acid and 2mM β-glycerophosphate) for two weeks and medium was changed every two to three days. Cells were then lysed using TRIzol reagents. Total RNA was extracted as per the manufacturer’s protocol. RNA was quantified using a NanoDrop® ND-1000 Spectophotometer and RNA quality was checked using an Agilent 2100 Bioanalyzer. Only the RNA samples with integrity number (RIN) higher than 9.8 were selected to carry on first-strand cDNA synthesis. To synthesise the cDNA, Promega ImProm-II™ reverse trancriptase and Oligo(dT) primer were used according to the manufacturer’s instructions. Reverse transcription (RT)-PCR was performed with primer sets specific for the various P2X7 receptor transcripts. Forward primers to determine a variant was 5'-CACATGATCGTCTTTTCCTAC-3' and for k variant was 5'-GCCCGTGAGCCACTTATGC-3’ in exon 1. Either of these were combined with the reverse primer in exon 4 5’-GGTCAGAAGAGCACTGTGC-3' and cycling conditions were 94°C for 2 min, followed by 35 cycles of 94°C for 40 s, 58°C for 40 s, 72°C for 40 s with a final extension step of 72°C for 10 min. The PCR products were resolved using 1% agarose gel and visualized by ethidium bromide staining using Bio-Rad GelDoc ™ XR+ Gel imaging system with a Quantity One software.
